# Scaffolds obtained from decellularized human extrahepatic bile ducts support organoids to establish functional biliary tissue in a dish

**DOI:** 10.1002/bit.27613

**Published:** 2020-11-09

**Authors:** Jorke Willemse, Floris J. M. Roos, Iris J. Voogt, Ivo J. Schurink, Marcel Bijvelds, Hugo R. de Jonge, Luc J. W. van der Laan, Jeroen de Jonge, Monique M. A. Verstegen

**Affiliations:** ^1^ Department of Surgery Erasmus MC‐University Medical Center Rotterdam The Netherlands; ^2^ Department of Gastroenterology and Hepatology Erasmus MC‐University Medical Center Rotterdam The Netherlands

**Keywords:** personalized regenerative medicine, LGR5^+^ organoids, extrahepatic bile duct, decellularization, tissue engineering

## Abstract

Biliary disorders can lead to life‐threatening disease and are also a challenging complication of liver transplantation. As there are limited treatment options, tissue engineered bile ducts could be employed to replace or repair damaged bile ducts. We explored how these constructs can be created by seeding hepatobiliary LGR5^+^ organoids onto tissue‐specific scaffold. For this, we decellularized discarded human extrahepatic bile ducts (EBD) that we recellularized with organoids of different origin, that is, liver biopsies, extrahepatic bile duct biopsies, and bile samples. Here, we demonstrate efficient decellularization of EBD tissue. Recellularization of the EBD extracellular matrix (ECM) with the organoids of extrahepatic origin (EBD tissue and bile derived organoids) showed more profound repopulation of the ductal ECM when compared with liver tissue (intrahepatic bile duct) derived organoids. The bile duct constructs that were repopulated with extrahepatic organoids expressed mature cholangiocyte‐markers and had increased electrical resistance, indicating restoration of the barrier function. Therefore, the organoids of extrahepatic sources are identified to be the optimal candidate for the development of personalized tissue engineered EBD constructs.

## Graphical abstract

Willemse and coworkers successfully decellularized human extrahepatic bile duct tissue. Organoids from three different hepatobiliary sources (intrahepatic bile ducts, extrahepatic bile ducts, and bile) were tested for their repopulation efficiency on the decellularized ductal matrix. Bile‐ and extrahepatic bile duct‐derived organoids were capable of repopulating the ductal matrix, expressed cholangiocyte‐specific markers, and restored barrier function.

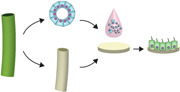

## 1 INTRODUCTION

Biliary complications, such as bile leaks and anastomotic strictures, occurring after donation after circulatory death (DCD) liver transplantation (LT) are common and a major cause of posttransplant complications and morbidity (Hessheimer et al., 2016; de Vries et al., 2018). In DCD LT, the biliary epithelial lining of the extrahepatic bile ducts (EBD) is more prone to damage, due to the prolonged warm ischemia period in the donor. This results in a higher incidence of ischemia type biliary lesions, which leads to more diffuse nonanastomotic biliary strictures, when compared with donation after brain death (16% vs. 3%; Blok et al., 2016; Foley et al., 2011). Current treatment options, such as endoscopic retrograde cholangiopancreatography and hepaticojejunostomy, often fail to restore biliary drainage and up to 65% of patients with ischemic cholangiopathy after LT require retransplantation (Foley et al., 2011). Personalized regenerative medicine strategies could prevent the need for retransplantation of the whole liver in case the donor liver is failing due to aforementioned complications. Damaged extrahepatic bile duct (EBD) can be replaced with functional tissue engineered constructs, preferably built using the autologous cells. These strategies can potentially relief the intense pressure on the already limited donor organ pool.

The EBD is not merely a “simple tube” that transports cytotoxic bile, as the EBD contains complex tortuous networks of peribiliary glands (PBG) and blood vessels. The PBG can be found in‐ (intramural) and outside (extramural) the wall of the EBD and play an important role in maintaining homeostasis and bile duct regeneration after injury (de Jong et al., 2018). The PBG are surrounded by small blood vessels, which are also known as the peribiliary vascular plexus. Recreating these small glandular and tortuous structures in vitro with high precision is challenging. Therefore, the use of decellularized extracellular matrix (ECM) could be an interesting alternative for recreating EBD tissue in vitro, where the decellularized ECM functions as a scaffold or the EBD (Crapo et al., 2011).

The decellularized scaffolds need to be repopulated with biliary cells to restore the vital barrier function of the bile duct against the cytotoxic bile. Human LGR5^+^ biliary organoids are an interesting source of functional biliary cells, as organoids offer long‐term stable in vitro expansion of cholangiocytes (Aloia et al., 2019; Huch et al., 2015). This allows for the generation of large numbers of autologous cells in vitro from relative small (liquid) biopsy samples.

The organoid cultures can be established from liver biopsies (intrahepatic bile duct‐derived organoids [IDO]; Huch et al., 2015), EBD tissue (extrahepatic bile duct‐derived organoids [EDO]; Rimland et al., 2020; Sampaziotis et al., 2017), and bile samples (bile‐derived organoids [BDO]; Soroka et al., 2018). The cells that give rise to these biliary organoids are EPCAM positive and organoids from all three sources maintain cholangiocyte‐specific markers (e.g., EPCAM, cytokeratin 7 [KRT‐7] or cytokeratin 19 [KRT‐19]) and functionality (Aizarani et al., 2019; Aloia et al., 2019; Huch et al., 2015; Rimland et al., 2020; Sampaziotis et al., 2017; Soroka et al., 2018). Furthermore, relative small biopsies (0.5–1.0 g tissue or 1 ml of bile) are adequate to initiate cultures, which subsequently can yield millions of cells (Schneeberger et al., 2020; Willemse et al., 2017). These characteristics make the organoids ideal cell sources for the repopulation of decellularized EBD scaffolds in an effort to create functional tissue engineered bile ducts in vitro. However, whether biliary organoids from all three sources are capable of efficient repopulation and restore the vital barrier function of the EBD, is yet to be determined.

Therefore, we aimed to develop an in vitro model for bile duct tissue engineering in which the recellularization capacity and, moreover, bile duct functionality after recellularization of the biliary organoids collected from the three sources, can be assessed. We first developed an efficient decellularization protocol for human EBD tissue to obtain ductal ECM. Subsequently, IDO, EDO, and BDO were expanded and used to recellularize the decellularized EBD scaffold. Confluency was used as a measure for the seeding efficiency of the epithelial monolayer. Furthermore, we analyzed expression of cholangiocyte markers and tested biliary function of the tissue engineered constructs.

## 2 METHODS

### 2.1 Sample procurement

#### 2.1.1 Sample procurement for decellularization

Biopsies of healthy EBD tissue (*N* = 26) were collected during LT. Before transplantation, the duct of the donor organ is shortened to make the anastomosis with the recipients' bile duct. The removed section (usually 3–10 mm) was stored in Belzer UW cold storage solution (UW; Bridge to Life) at 4°C. Four segments (3–5 mm by 3–5 mm) were cut from the biopsy. One segment was used for organoid initiation (see sample procurement for organoid initiation, *N* = 5). The second segment was fixed in 4% paraformaldehyde (PFA; Fresenius Kabi) for histological analysis. The two other segments were snap frozen in liquid nitrogen and stored at −80°C for biochemical analysis purposes. The remaining EBD tissue was stored in 1× phosphate‐buffered saline (PBS) at −20°C until decellularization. The use of these biopsies for research purposes was approved by the Medical Ethical Council or the Erasmus University Medical center (MEC‐2014‐060) and patients gave their written informed consent.

Full length EBD tissue (average length: 3–5 cm, *N* = 8) was obtained from human research livers. These livers were deemed unsuitable for clinical transplant procedures in the EuroTransplant zone by all transplant centers, due to a variety of reasons, such as steatosis and/or age (*N* = 8). No organ retrieval was initiated for research purposes only. In all cases, next of kin gave informed consent for research to Transplant Coordinators of the Dutch Transplantation Society (NTS). The use of research liver was approved by the Erasmus MC medical ethics committee (MEC‐2012‐090). After organ procurement, the liver was stored in UW organ preservation fluid (Bridge to life) on ice and shipped to the Erasmus MC, where the EBD was surgically removed from the liver. Small biopsy samples were taken in a similar manner as previously described, except for organoid initiation. The remaining EBD tissue was placed in PBS and stored at −20°C.

#### 2.1.2 Sample procurement for organoid initiation

Biopsies of liver tissue (0.5–2 cm^3^; *N* = 5) and EBD tissue (*N* = 5) were obtained for organoid initiation during LT at the Erasmus University Medical Center Rotterdam from healthy donor tissue (*N* = 3) and diseased explant tissue (*N* = 2; recurrent primary sclerosing cholangitis and Wilson's disease). Liver and EBD biopsies were donor or patient paired. The use of these biopsies for research purposes was approved by the Medical Ethical Council or the Erasmus University Medical Center (MEC‐2014‐060) and written informed consent was given by the next of kin of the donor or by patients. Biopsies were stored and transported in UW preservation fluid on ice.

Bile (1 ml) was collected from patients (*N* = 3) undergoing ERCP procedures during treatment for primary sclerosing cholangitis, bile leakage, or choledocholithiasis. The use of bile samples for research purposes was approved by the Medical Ethical Council of the Erasmus University Medical Center (MEC‐2016‐743). All patients gave written informed consent. Samples were stored and transported on wet ice.

### 2.2 Decellularization

The EBD was washed with dH_2_O until all traces of blood or bile were removed from the EBD. The lumen of the full length EBD was flushed using a blunt needle. Subsequently, the ductal tissue was incubated with Trypsin‐EDTA (TE) (0.05%, Gibco) for 30 min at 37°C on an orbital shaker. TE was washed away with dH_2_O for 15 min. Subsequently, the EBD was placed in 50 ml of 4% Triton‐X‐100 + 1%NH_3_ (T×100 solution) on an orbital shaker at room temperature (RT). T×100 solution was replaced every 30 min until 10 cycles were reached. The EBD tissue was placed in 50 ml dH_2_O for 5 min and dH_2_O was refreshed 10 times. The decellularized EBD tissue was stored in 50 ml dH_2_O at 4°C for 5–7 days to remove traces of T×100. dH_2_O was refreshed every 1 or 2 days.

The decellularized duct was incubated with DNase solution (Table S1) for 4 h at 37°C on an orbital shaker. Afterwards, the EBD was placed in 50 ml 0.9% saline solution, which was refreshed three times. Biopsy samples were taken for histological and DNA analysis.

#### 2.2.1 Histology

PFA‐fixed samples were embedded in paraffin and sectioned at 4 µm. Sections of before and after decellularization samples were stained with hematoxylin and eosin (H&E) or 4′,6‐diamidino‐2‐phenylindole (DAPI; Vectashield, Vectorlabs). H&E stained slides were imaged with Zeiss Axiokop 20 microscope and captured with a Nikon DS‐U1 camera. DAPI stained slides were analyzed using EVOS microscope (Thermo Fisher Scientific).

Immunohistochemistry (IHC) staining was performed on before and after decellularization samples with Collagen Type I and Collagen type IV (Table S4). Antigen retrieval was performed in citrate buffer (pH = 6.0) at subboiling temperatures for 10 min. Primary antibodies were incubated over night at 4°C. Envision + system horseradish peroxidase antirabbit secondary antibody (DAKO) was incubated at RT for 60 min, before staining with 3'‐diaminobenzidine and and counterstaining with hematoxylin.

#### 2.2.2 Scanning electron microscopy

Small PFA‐fixed biopsies (before and after decellularization, *N* = 2) were dehydrated with ethanol and hexamethyldisilazane (Sigma) series before gold sputtering (15 µm) using a Quorum Q300T D sputtering device (Quorumtech). Biopsies were imaged with a JSM‐7500F field emission electron microscope (JEOL).

#### 2.2.3 Biochemical analysis

The wet weight of the samples was weighed before performing analysis.

DNA was isolated using a QIAamp DNA mini Kit (Qiagen) following the manufacturer's protocol. DNA content was measured using a NanoDrop spectrophotometer (Thermo Fisher Scientific; *N* = 11) and corrected for the corresponding wet weight of the measured sample (ng DNA/mg wet weight tissue). The quality and length of DNA base pairs (BP) was measured using a 2100 BioAnalyzer (Agilent technologies) using a DNA‐1000 kit (Agilent Technologies).

Total collagen content of the samples was determined using a Total Collagen Kit (Quickzyme Biosciences, *N* = 9). Collagen content was measured in a clear 96‐well plate at 570 nm using an Omega POLARstar Microplate reader (BMG labtech). The content was corrected for the wet weight of the corresponding samples (µg Collagen/mg wet weight tissue).

Sulfated glycosaminoglycan (sGAG) was determined using a Blyscan glycosaminoglycan assay (Biocolor; *N* = 15). Samples were digested in a Papain (Sigma) solution (10 mg/ml) at 65°C for 8 h. sGAG was isolated from the sample digest according the manufacturers protocol. The sGAG content was measured by absorbance measurements (680 nm) in a clear 96‐well plate using a model 680 XR microplate reader (Bio‐Rad).

### 2.3 Recellularization

#### 2.3.1 Initiation tissue derived organoids (IDO and EDO)

**Figure 1:**
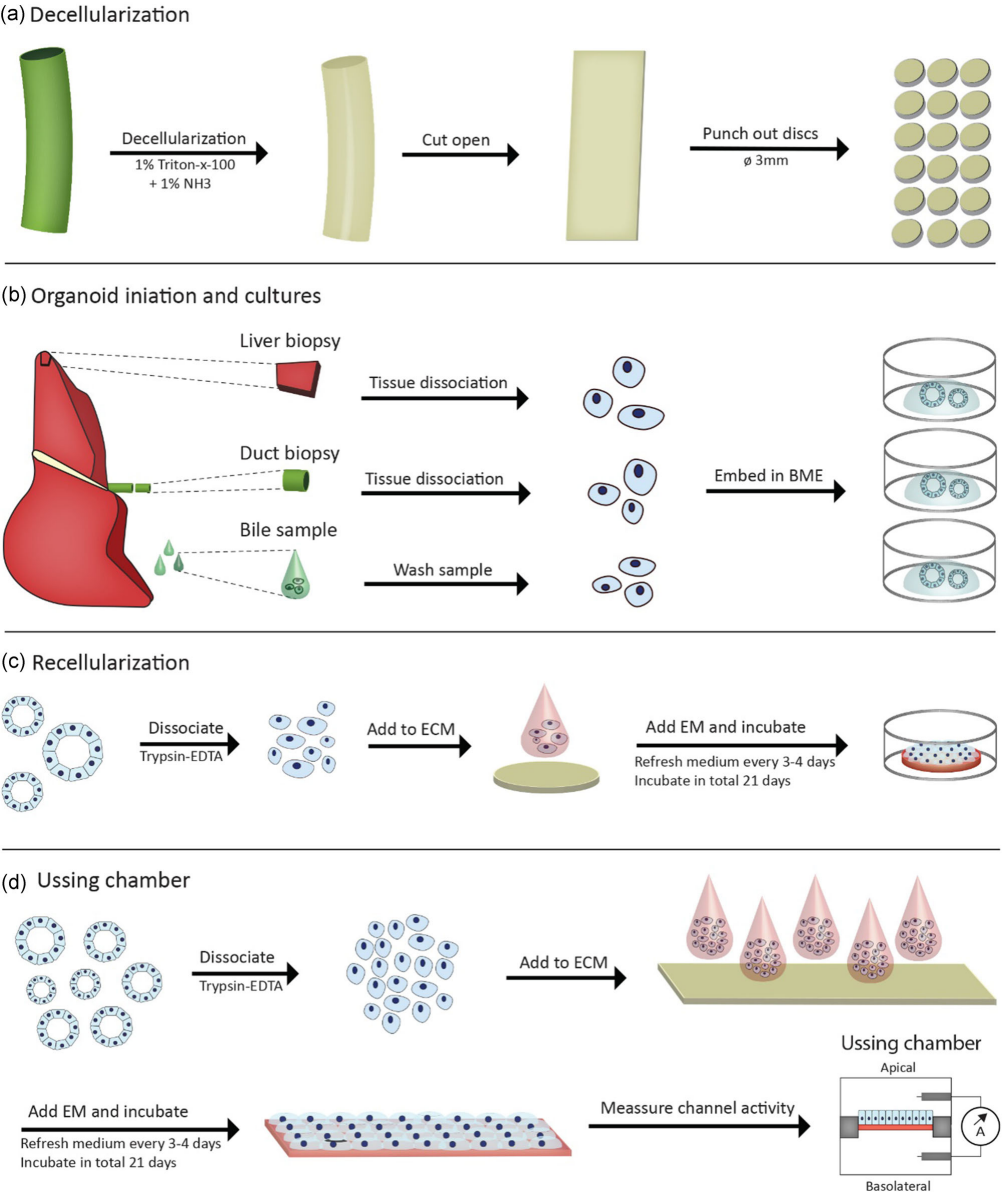
Graphical representation of human EBD decellularization and recellularization procedures. (a) Decellularization of EBD tissue was performed with T×100 solution. After decellularization the full length EBD was cut open along the longitudinal axis and circular discs (Ø3 mm) are punched using a dermal biopsy punch. (b) Organoids were initiated from three different sources; liver tissue (IDO), EBD tissue (EDO), and bile samples (BDO). The cells obtained from these sources were embedded in BME and cultured as per normal protocol. (c) Recellularization experiments start with dissociation of the organoids. A suspension of single cells (10 µl) was added to the ECM and kept in culture for up to 21 days. (d) Recellularization experiments for Ussing Chamber were performed in a similar manner as the normal recellularization experiments. After a 21‐day culture period, the recellularized construct was carefully placed inside the Ussing chamber setup, followed TEER and ion‐channel activity measurements in Ussing chambers. BDO, bile‐derived organoids; BME, basement membrane matrix; EBD, extrahepatic bile duct; ECM, extracellular matrix; EDO, extrahepatic bile duct‐derived organoids; IDO, intrahepatic bile duct‐derived organoids; TEER, trans epithelial electrical resistance [Color figure can be viewed at wileyonlinelibrary.com]

Organoid initiation was similar as previously described (IDO; Huch et al., 2015; EDO; Rimland et al., 2020). See Figure 1b for a schematic overview of organoid initiation and the section “Sample procurement for organoid initiation” for more details on tissue procurement. In short, biopsies were minced, digested in 2.5 mg/ml collagenase type A (Sigma) for 20 min at 37°C. The cell suspension was strained (70 µm cell strainer) and washed in cold Advanced DMEM/F12 (Adv+, Table S2). After centrifugation (1500RPM, 5 min, 4°C) the remaining cell pellet was suspended in reduced growth factor basement membrane matrix (BME, Cultrex) solution (70% BME, 30% cold Adv+). The mixture was plated in 25 µl droplets in a 48‐well suspension culture plate (Greiner). The BME solidified at 37°C for 30–45 min before startup medium (SM, Table S3) was added. After 3 days SM was replaced with expansion medium (EM, Table S3).

#### 2.3.2 Initiation bile‐derived organoids

The obtained bile was suspended in 8 ml cold Adv+, centrifuged (1500RPM, 5 min, 4°C) and the supernatant was removed. This procedure was repeated once. The remaining cell pellet was suspended in 3 ml cold Adv+, strained (100 µm cell strainer) and centrifuged. The cell pellet was suspended in 70% BME solution and cells were treated similar to IDO and EDO.

#### 2.3.3 Culturing organoids

EM was refreshed of all three types of organoids every 3 to 4 days. Organoids were split in 1:4 to 1:6 ratios every 7 to 10 days depending on proliferation rate of the cells by mechanical dissociation and replating of organoids fragments in fresh BME.

#### 2.3.4 Preparation of the ECM

Full length decellularized EBD was cut open along the longitudinal axis (Figure 1a). Circular discs (Ø 3 mm) were cut using disposable dermal biopsy punches (Stiefel). The discs were collected in 50 ml 1× PBS and washed in PBS three times. This was repeated with Adv+ and with Adv+ supplements with 10× concentration Pen/Strep and primocin. The discs were incubated overnight at 37°C. The discs were washed in Adv+. The ECM was placed in the middle of a 48‐well 45 min before addition of cells. Residual adv+ was removed from the surface and side of the discs.

#### 2.3.5 Recellularization experiments

Organoids were harvested by removing the BME droplets from the wells using cold adv+, as previously described (see Figure 1c for a schematic overview). In general, a full BME droplet (average yield: 6.0 × 10^4^ cells, *SD*:±2.0 × 10^4^ cells per dome) was used per ECM disc. After removal of BME from the cell pellet, 1 ml TE was added. The suspension was incubated at 37°C until organoid fragments were dissociated into a single cell suspension. The cells were counted using disposable cell counting chambers (Kova). About 10 µl cell suspension was added to the center of the ECM discs.

The samples were incubated at 37°C for 2 h before 500 µl EM supplemented with 10 µM Y27632 was added to the wells. EM+Y27632 was replaced with EM after 3 days and medium was refreshed every 3 or 4 days. The ECM‐cell construct was kept in culture for up to 21 days. Organoid cultures in BME served as a control.

After 21 days experiments were terminated. About 4–6 samples were fixed in 4% PFA for 20 min. These samples were used for histological analysis or whole mount staining. These 4–6 samples were lyzed in 700ul Qiazol lysis reagent (Qiagen) and stored at −80°C for quantitative polymerase chain reaction (qPCR) analysis.

#### 2.3.6 Ussing chamber experiments

Larger segments (W: 1 cm, L: 2 cm) of ECM were cut from the EBD using a scalpel. The recellularization procedure was similar to the circular discs recellularization, however, the cell number was increased five‐fold (see Figure 1d for a schematic overview). About 5×10 µl cell suspension droplets were used for each segment. Furthermore, EM was refreshed every 1–2 days. After 21 days the segment was cut in two equal sized parts. Each part was placed in an Ussing slider (P2303A, area: 0.10 cm^2^, Physiologic Instruments, Figure S2) and subsequently placed in the Ussing chamber (Physiologic Instruments). Decellularized ECM was used as a control for the Ussing chamber experiments. The Ussing chambers were filled with Meyler's medium (Table S7) supplemented with 10 mM glucose. The Ussing chambers were kept at 37°C and a 95% O_2_ 5% CO_2_ gas mixture was bubbled through the chambers. A VCC MC8 voltage clamp module (Physiologic Instruments) was used to clamp the potential difference at 0 mV. The short circuit current (*I*_sc_) was recorded using Acquire and Analyze 2.3 software (Physiologic Instruments). Trans epithelial electrical resistance (TEER) measurements was measured by applying three 5 V spikes. The resistance was calculated according to Ohm's law

(1) R=V/I

The resistance of the recellularized constructs was calculated by subtracting measured resistance value of decellularized ECM

(2) $$R_{organoids}=R_{constructs}-R_{decellularized\;ECM}$$

*R*_organoids_ was subsequently corrected for the surface area (A = 0.10 cm^2^) of the Ussing slide

(3) $$TEER=R_{organoids}\cdot A_{tissueslide}$$

Subsequently, ion‐channel activity was measured. CFTR‐dependent anion secretion was activated by adding forskolin (10 µM) to the basolateral side of the constructs and inhibited by addition of GlyH‐101 (20 µM, apical). The calcium activated chloride channels (CaCC) were stimulated by addition of UTP (50 µM, apical).

#### 2.3.7 Immunohistochemistry of organoids

Organoids were cultured in BME, fixed in 4% PFA and embedded in paraffin. Subsequently, IHC staining was performed as described previously for the IHC procedure in the histology Section (2.2.1). The primary antibodies (Cytokeratin 7 (KRT‐7) and cytokeratin 19 (KRT‐19; Table S4) were incubated overnight at 4°C. The secondary antibody (Table S5) was incubated at RT for 60 min.

Whole mount confocal imaging was performed on 4% PFA fixed recellularized scaffolds. Recellularized ECM samples were permeabilized with 0.1% Triton‐X‐100 in 1× PBS for 20 min. The samples were blocked in 5% serum in 1× PBS for 60 min. The primary antibodies (see Table S4) were incubated overnight at 4°C. The secondary antibody (Table S5) was incubated at RT for 60 min. KRT‐7 and KRT‐19 samples were additionally stained with Phalloidin Alexa Fluor 488 (Thermo Fisher Scientific). All samples were stained with DNA‐staining DAPI. Samples were imaged using a Leica ×20 water dipping lens on Leica DM6000 CFS microscope with a LEICA TCS SP5 II confocal system. Images were processed and analyzed using ImageJ.

#### 2.3.8 qPCR gene expression analysis

Qiazol lyzed samples were homogenized using a TissueRuptor (Qiagen). Messenger RNA (mRNA) isolation was performed with the miRNeasy kit according to the manufacturers' protocol. RNA content was measured using a Nanodrop and complementary DNA (500 ng) was prepared using 5× PrimeScript RT Master Mix and a 2720 thermal cycler (Applied Biosystems). qPCR was performed according to standard procedures with SYBR select master mix for SFX (Applied Biosystems) on a StepOnePlus real time PCR System (Applied Biosystems). All the tested primer sets are listed in Table S6.

GAPDH, B2M, and HPRT were used as reference genes. The geometrical average of the three housekeeping genes was used as previously described (Vandesompele et al., 2002) for determining the dCt of the genes.

### 2.4 Data analysis

Analysis of data was performed with Prism (version 8.0, Graphpad Software). Data from DNA, RNA, total collagen, sGAG content, and Nuclei per mm^2^ is displayed as mean ± standard deviation (*SD*). Nonpaired *t*‐test were performed to analyze means. Analysis of variance on ranks was performed for the quantified nuclei data. qPCR data is displayed as 2^−dCt^ in “before–after” graphs, were “before” represents the BME controls and “after” the recellularized constructs of the same donor/patient. Wilcoxon matched pairs tests were performed on qPCR data.

## 3 RESULTS

**Figure 2:**
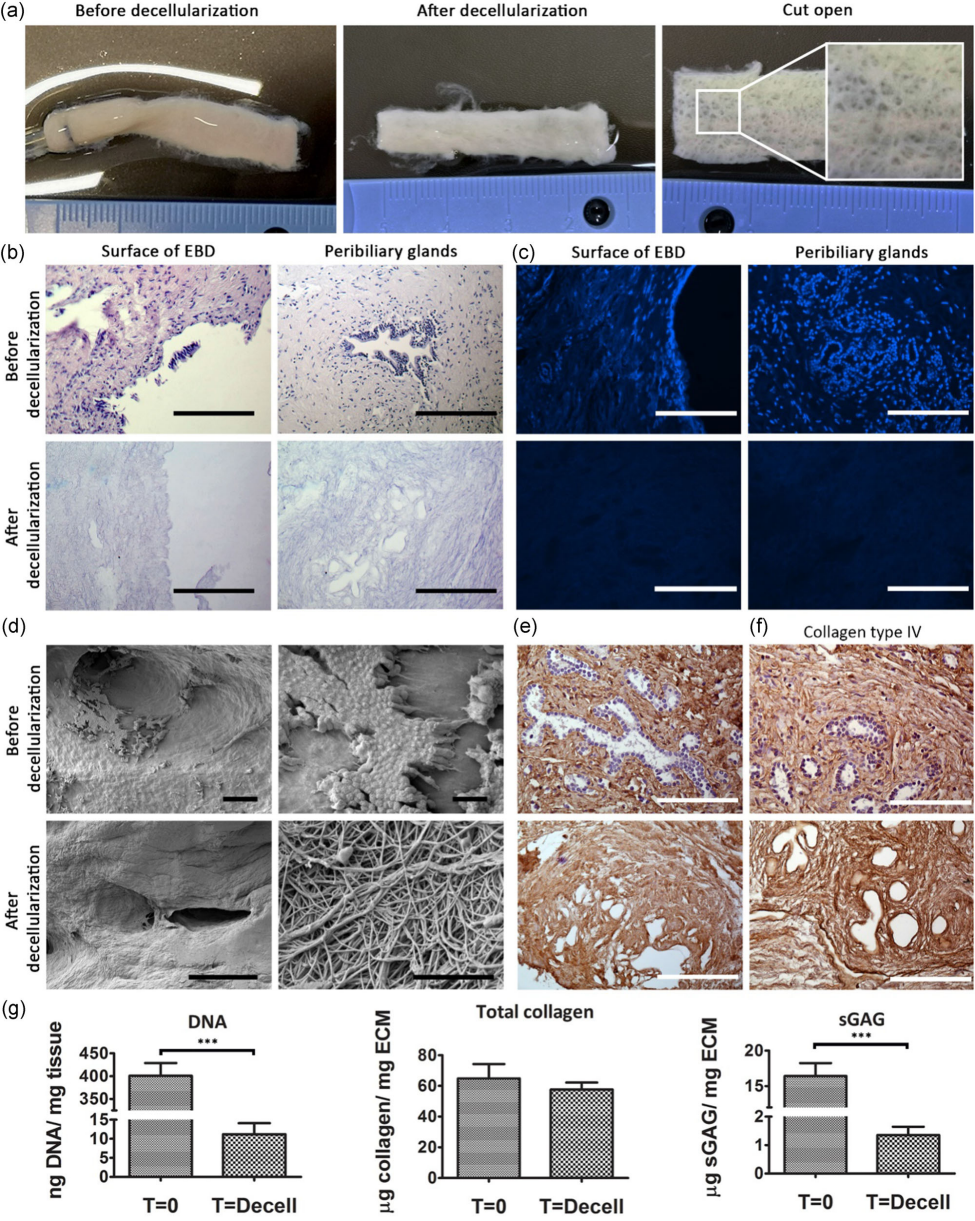
Decellularization of human extrahepatic bile duct tissue is feasible. (a) Macroscopic images of before and after decellularization. The ostia of the PBG are visible (outtake). (b) Before and after decellularization images of H&E stained paraffin slides showing the surface of the EBD (left) and intramural PBG (right). Scale bars represent: 200 µm. (c) DAPI staining revealed that after decellularization no visible dsDNA is present in the decellularized ECM on the surface of the EBD or in the PBGS. Scale bars represent: 200 µm. (d) Scanning electron microscopy images before decellularization shows the expected denudation of the extrahepatic bile duct as only a few cells can be seen at two different magnifications from the same sample. After decellularization all cells were removed and the ultrastructure of the ECM remains intact as is shown by two different magnifications from the same sample. Scale bars represent 200 µm left side, 10 µm top right, 1 µm bottom right. (e) Collagen Type I remains present after decellularization. Scale bars represent: 200 µm. (f) Collagen type IV remains present in the PBG after decellularization. Scale bar represents: 200 µm. (g) Results from the biochemical analyses. DNA drops significantly (***p < .001) after decellularization to 11.9 ng DNA (*SD* ± 9.2 ng DNA). Total collagen content does not differ significantly (*N* = 9). sGAG content decreases significantly (***p < .001) to 1.4 µg sGAG/mg ECM (*SD*± 1.0 µg sGAG; *N* = 15). DAPI, 4′,6‐diamidino‐2‐phenylindole; EBD, extrahepatic bile duct; ECM, extracellular matrix; H&E, hematoxylin and eosin; PBG, peribiliary glands; sGAG, sulfated glycosaminoglycan [Color figure can be viewed at wileyonlinelibrary.com]

All EBD samples showed severe signs of denudation before decellularization, as no confluent layers of cholangiocytes could be found (Figures 2b and 2d). This was likely the result of ischemia. Due to the denudation, no cholangiocyte RNA of adequate quality could be obtained from fresh EBD tissue. During decellularization, the bile ducts underwent a slight change in color from yellow/white to white (Figure 2a). The decellularization procedure did not affect the dimensions of the EBD, as no shrinking or expansion was witnessed (Figures 2a and S1). However, loose connective tissue fibers surrounding the bile ducts (Figures 2a and S1), detached as a result of gentle agitation on the rocker and was washed away during replacement of T×100 solution. This connective tissue did not contain muscle, extramural PBG or blood vessel structures. Full length EBDs were cut open along the longitudinal axis after the decellularization procedure was completed and showed typical “golf ball‐like” surface macroscopically (Figure 2a). The decellularization procedure did not affect the macroscopic architecture of the luminal side of the duct.

All cells were efficiently removed during the decellularization procedure (Figure 2b) and no dsDNA (Figure 2c) was detected in the decellularized EBD. DNA quantification (Figure 2g) showed a significant (*p* < .001) decrease in DNA content to 11.9 ng DNA (*SD*:±9.2 ng). No fragment of dsDNA could be detected by the BioAnalyzer after decellularization (Figure S1), confirming complete decellularization. The fibrous ultrastructure of the ECM remained intact and was not affected by the procedure as shown by Scanning electron microscopy (SEM; Figure 2d). IHC staining of the decellularized bile ducts for Collagen I and IV showed that these proteins remain present (Figure 2e,f). The total collagen content did not differ significantly (*p* = .69) before (64.9 µg/mg wet tissue, *SD*:±21.0 µg/mg) and after decellularization (57.6 µg/mg wet ECM, *SD*:±12.9 µg/mg). The sGAG content decreased significantly (*p* < .001) after decellularization (before: 16.4 µg/mg wet tissue, *SD*: ±6.8 µg/mg; after: 1.4 µg/mg wet ECM, *SD*: ±1.1 µg/mg), which could be due to the detachment and subsequent removal of connective tissue on the outside of the ductal tissue, however, this was not further determined.

Organoids from all three ductal sources proliferated well, were spherical in shape (Figure S2) and were comparable with organoids as previously described by other publications (Huch et al., 2015; Rimland et al., 2020; Sampaziotis et al., 2017; Soroka et al., 2018). The organoids were KRT‐7 and KRT‐19 positive (Figure S2). Differences in proliferation patterns were noticed, however, these were attributed to donor‐donor variances, as patient/donor paired organoids showed similar characteristics (data not shown). Similar findings were also mentioned by other publications (Huch et al., 2015). Furthermore, no significant differences were noted between organoids derived from healthy donor or liver patients, as all organoids were similar in size, shape or proliferation patterns.

**Figure 3:**
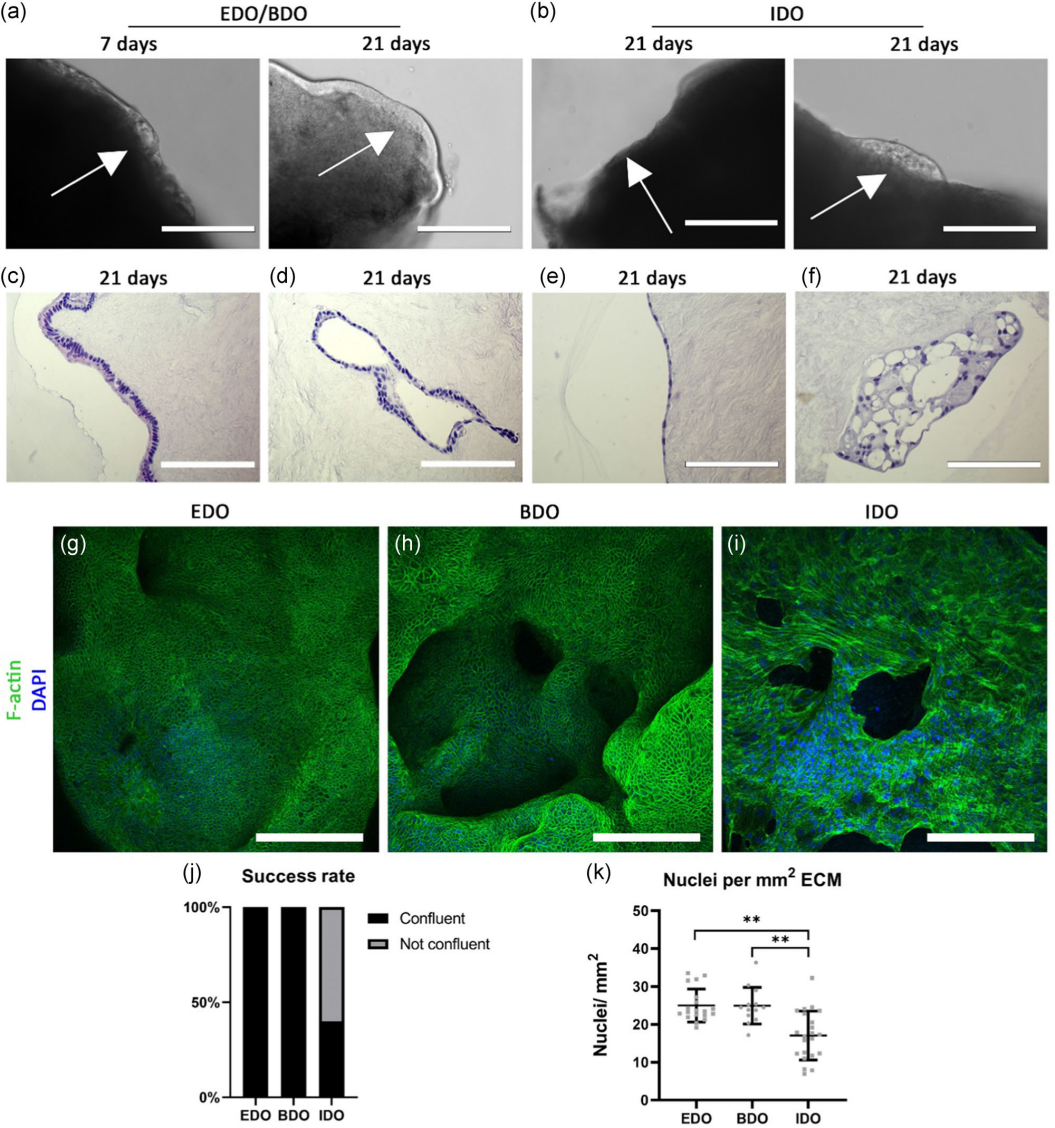
EDO and BDO can fully repopulate the luminal surface of ductal ECM. (a) Decellularized ECM were not transparent. Only cells surrounding the edges of the scaffold can be monitored. After 24 h cells can be found surrounding the ECM of EDO and BDO recellularized samples. After 7 days a semitransparent rim appeared, which contained cystic structures (white arrow). After 21 days no cystic structures could be detected (scale bars: 200 µm). (b) IDO were less efficient in recellularization of ductal ECM, as they formed thin edges surrounding the ECM. IDO showed either a cystic‐phenotype or flattened phenotype after 21‐days (scale bars: 200 µm). (c) EDO and BDO that encapsulated the luminal surface of the ECM had a columnar phenotype, where the nucleus was located towards the apical side of the ECM. (d) Cells were found inside the lumen of the ostia of the PBG. (e) IDO lack the columnar phenotype. Cells are flattened in shape. (f) In some cases, where IDO fail to recellularize the ductal ECM, cystic structures are seen after 21 days. Scale bars (c–f) 200 µm. (g–i) F‐actin staining on whole mounted samples. (g and h) EDO and BDO are capable of forming confluent layers on the luminal surface of the ductal ECM. Ostia of PBG (h) are also repopulated. (i) IDO expresses a different phenotype, as recellularization was not completed. Scale bar GHI: 200 µm. (j) The “success rate” as determined by the percentage of completely repopulated samples. For EDO and BDO the success rate was 100% as organoids from each donor or patient formed confluent layers (*N* = 5 EDO and *N* = 3 BDO). For IDO this percentage was 40%, as organoids from only two donors or patients were capable of forming confluent layers. The other three donor or patient IDO formed flattened layers with holes. (k) The amount of nuclei counted per mm^2^. EDO and BDO have 24.9 nuclei (*SD*:±4.2) and 25.0 nuclei (*SD*:±4.6) per mm^2^ respectively. The nuclear density of IDO differs significantly with EDO and BDO repopulated samples (**p < .01). IDO repopulated samples contained 17.1 nuclei per mm^2^ (*SD*:±6.3). Success rate (j) and nuclear density (k) were determined by examination and analysis of five whole mount confocal Z‐stacks of each source (*N* = 4 EDO and IDO, *N* = 3 BDO). BDO, bile‐derived organoids; ECM, extracellular matrix; EDO, extrahepatic bile duct‐derived organoids; IDO, intrahepatic bile duct‐derived organoids; PBG, peribiliary glands [Color figure can be viewed at wileyonlinelibrary.com]

**Figure 4:**
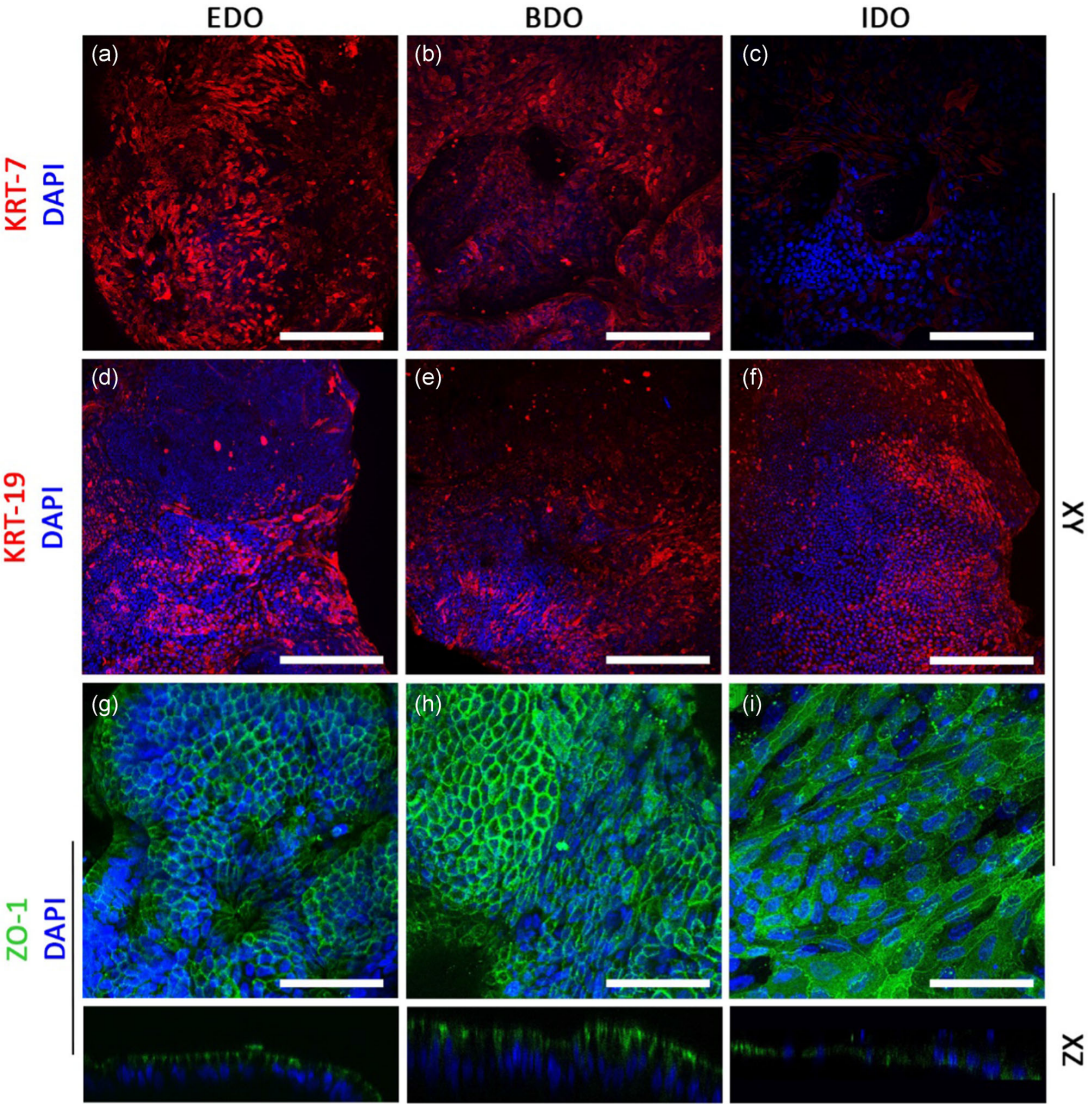
Organoids derived from EBD tissue or bile samples can be used to repopulate the apical surface of ductal ECM. (a–c) EDO and BDO express cytokeratin‐7 (KRT‐7), whereas IDO showed lower expression of KRT‐7. (d–f) cytokeratin‐19 (KRT‐19) expression was similar between EDO, BDO, and IDO. (k–m) Zone Occludens‐1 (ZO‐1) expression showed that EDO and BDO had a “honey comb”‐like phenotype. ZO1 was located on the luminal side (XZ plane), whereas nuclei were located at the basolateral side, indicating cholangiocyte‐like polarization of the cells. IDO recellularized samples had a flattened phenotype and no polarization of ZO‐1 was detected. Scale bars: (a–f) = 200 µm and (g–i) = 100 µm. BDO, bile‐derived organoids; ECM, extracellular matrix; EDO, extrahepatic bile duct‐derived organoids [Color figure can be viewed at wileyonlinelibrary.com]

Bright field microscopic evaluation of the recellularization experiments was limited as a result of the density of the ECM. However, viable cells surrounding the scaffolds were detected 24 h after initiating the recellularization with organoids from all three sources (Figure 3a). After 7–10 days transparent rim was seen surrounding the edge of the ECM disc in EDO and BDO recellularized samples. In IDO samples, this rim was inconsistent and did not fully cover the entire disc. Cyst‐like structures were seen inside the rim (Figure 4a). Between Day 15 and 18 these cystic structures disappeared in EDO and BDO samples and an uninterrupted rim encapsulated the edges of these samples after 21 days (Figure 3a). Cells with columnar phenotypes surrounded the ductal ECM (Figure 3c). This was not consistent with IDO recellularized samples, which had either cells with a flattened phenotypes or cystic structures after 21 days (Figures 3b and 3d).

Whole mount confocal analysis with F‐actin staining showed that confluent monolayers covered the entire surface of ECM discs recellularized with EDO and BDO (Figure 3g,h). “Honey comb”‐like structure were visible at ×40 magnification (Figure S2), showing that F‐actin is located at the edges of the cell membranes, where the cells attach to each other. ECM repopulated with IDO did not reveal similar patterns. Three out of five samples from this source failed to form a confluent layer on the entire surface of the ductal ECM (Figures 3i and S2). The two other samples lacked “honey comb”‐ like structures. The “success rate” as determined by the number of samples which were fully repopulated upon examination with whole mount confocal imaging is 40% for IDO (Figure 3j), whereas this rate was 100% for EDO and BDO. Quantification of nuclei per set area (Figure S2 contains representative images for all conditions) revealed that IDO repopulated samples contain significantly (*p* < .01) less nuclei per mm^2^ (17.1 nuclei per mm^2^, *SD*:±6.3) than EDO (24.9 nuclei per mm^2^, *SD*:±4.2) and BDO (25.0 nuclei per mm^2^, *SD*:±4.6). The difference between completely repopulated IDO samples (confluent, 20.5 nuclei per mm^2^, *SD*:±5.2) with the partially repopulated IDO samples (not confluent, 12.9 nuclei per mm^2^, *SD*:±4.8) was also significant (*p* < .01, Figure S2). Even after full repopulation with IDO, the number of nuclei per set area was lower when compared with samples repopulated with EDO and BDO, however, this difference is not significant.

**Figure 5:**
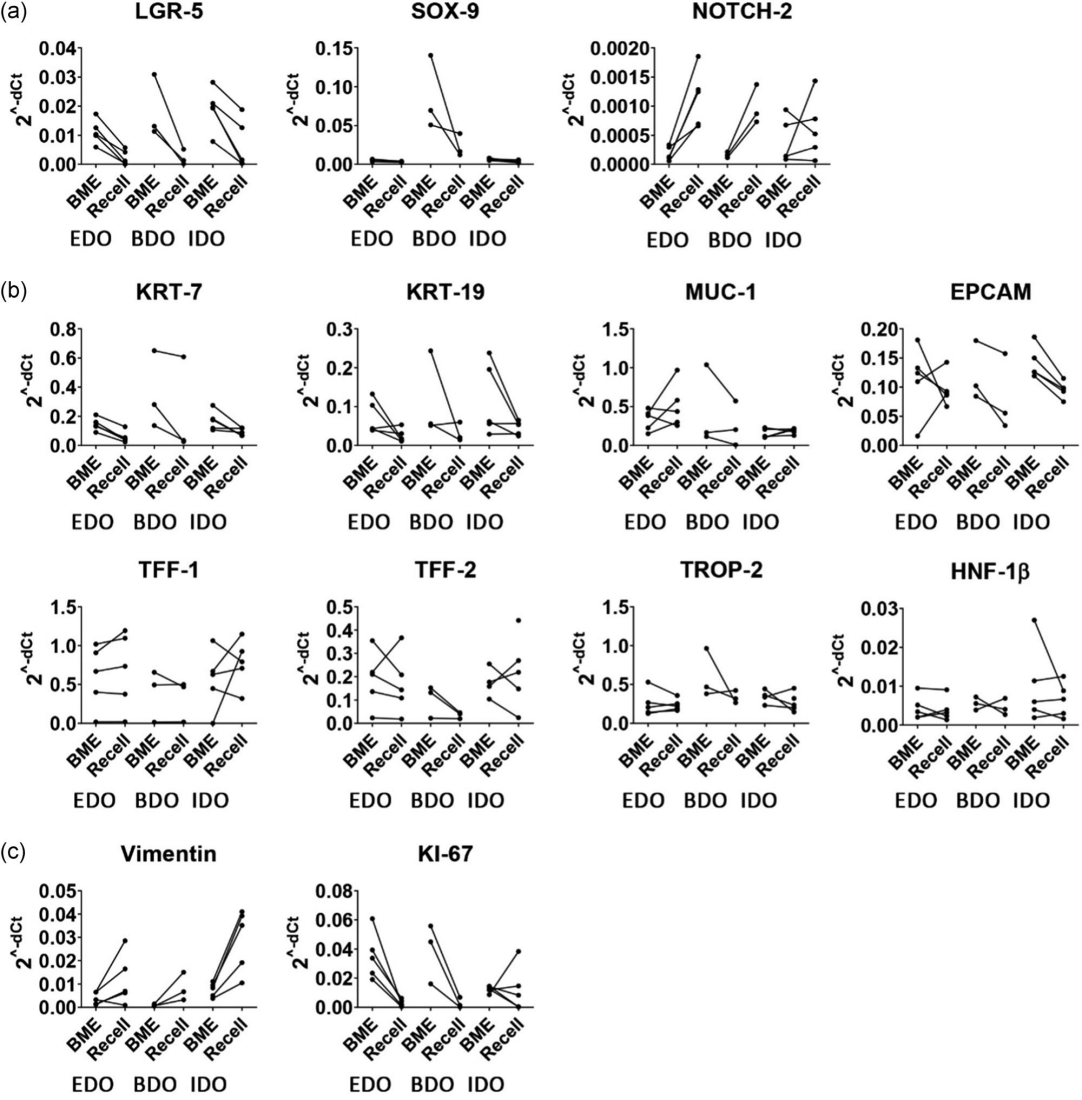
mRNA expression data before (BME) and after recellularization (Recell) on top of the ductal ECM of matched organoids from different origins. Cholangiocyte differentiation or stem cell markers are shown in panel (a). Cholangiocyte‐specific genes are shown in panel (b). Proliferation (KI‐67) and epithelial‐to‐mesenchymal transition marker (Vimentin) are displayed in panel (c). The RT‐qPCR data is displayed as 2^−dCt^. Wilcoxon signed rank test did not reveal any significant differences between the marched BME and recellularized samples. BME, basement membrane matrix; ECM, extracellular matrix; mRNA, messenger RNA; RT‐qPCR quantitative reverse‐transcription polymerase chain reaction

Expression of cholangiocyte marker cytokeratin‐7 (KRT‐7) differed between EDO, BDO and IDO repopulated ECM (Figure 4a–c), as KRT‐7 expression was lower in IDO samples compared with EDO and BDO. Cholangiocyte marker cytokeratin‐19 (KRT‐19) expression was similar for all repopulated ECM samples (Figure 4d–f). Protein expression of Zone Occludens‐1 (ZO‐1) was also seen in ECM samples recellularized with organoids from all three types, however, expression was seen in different patterns (Figure 5g–i). In EDO and BDO samples, ZO‐1 was located in between cells, showing that tight junctions formed between individual cells (Figure 4g,h). The XZ‐plane revealed that ZO‐1 expression was found at the luminal side, whereas nuclei are located at the basolateral side of the cells. This indicates cholangiocyte‐like polarization of the cells on EDO and BDO recellularized samples. Again, IDO showed a different pattern, as these cells lacked the “honey comb”‐like structured, had flattened phenotypes and no polarization was witnessed (Figure 4i)

Specific gene expression analysis showed a decrease in expression of LGR5 (Wnt target gene) after recellularization when compared with matched BME controls (Figure 5). IDO showed an approximate four‐fold decrease, whereas EDO had a 6.5‐fold and BDO a 10‐fold decrease after recellularization. SOX‐9 (biliary progenitor marker) expression of BDO samples (both BME controls and recellularized samples) was higher compared with SOX‐9 expression of EDO and IDO samples. Focusing on BDO samples only, the SOX‐9 expression decreased threefold after recellularization when compared with matched BME controls.

Expression of NOTCH‐2 was increased (approximately EDO: 3.5‐fold, BDO: 6.1 fold, and IDO: 3.1‐fold) after recellularization, indicating differentiation towards cholangiocytes. Interestingly, a decrease in KRT‐7 (approximately EDO: 8‐fold, BDO: 2.3 fold, and IDO: 1.6 fold decrease) and KRT‐19 (approximately EDO: 2.5‐fold, BDO: 2‐fold, and IDO: 1.7‐fold decrease) was measured, whereas KRT‐7 and KRT‐19 staining showed that on protein level these proteins are still expressed. Recently, it was shown that there is no significant differential gene expression of KRT‐7 and KRT‐19 between primary extrahepatic cholangiocytes and EDO (Rimland et al., 2020). Expression of mature cholangiocyte markers MUC‐1, TFF‐1, TFF‐2, EPCAM, TROP‐2, and HNF‐1β remained stable.

KI‐67 expression, as indicator for cell proliferation, decreased when recellularized with EDO and BDO, whereas in some of the IDO samples this was inconclusive. The IDO samples, which reached confluency had a decrease in KI‐67 expression (1.6‐fold, 33‐fold, and 50‐fold), whereas nonconfluent IDO samples had an increase (4.4 and 1.2 fold) in expression when compared with matched BME controls. In all cases, an increase in Vimentin expression was measured after recellularization (EDO: 3.8‐fold, BDO: 1.6‐fold, and IDO: 9.7‐fold increase) indicating that cells were undergoing epithelial‐to‐mesenchymal transition.

Expression of the cholangiocyte‐specific transporter and channel genes CFTR, SLC‐4a2, and SLC10a2 (ASBT) was also detected (Figure S3) suggesting that the cells could be capable of performing anion and bile salt transport functions. Recellularization on ECM discs did not affect expression of hepatocyte markers Albumin, CYP‐3a4, ABCB11 (BSEP), and HNF‐4α (Figure S3). No apparent hierarchical clustering could be found between recellularized samples or organoids from the same patients in BME. Similarly, no clustering could be found between organoids derived from healthy donors or patients (data not shown).

**Figure 6:**
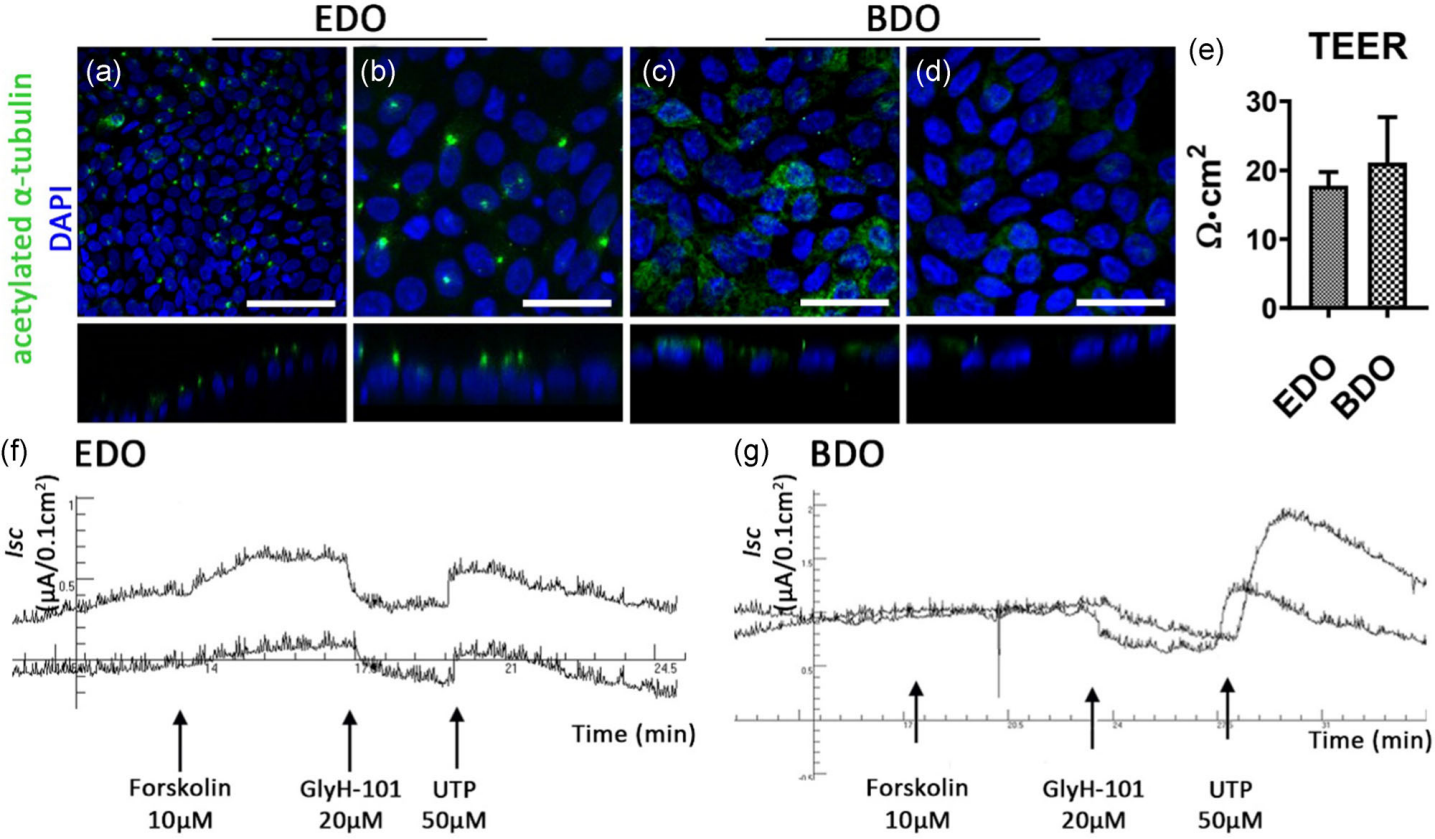
Functional bile duct constructs can be created in vitro using EDO or BDO. (a–d) Acetylated α‐tubulin staining shows the presence of cilia in EDO and BDO recellularized samples. Scale bars: (a) 100 µm, (b–d): 50 µm. (e) TEER measurements show an increase in resistance compared to decellularized ECM (*N* = 3 measurements per sample). (f) and (g): Ussing chamber experiments showing short circuit current (*I*_sc_) changes upon consecutive addition of Forskolin (a cAMP agonist), GlyH‐101 (CFTR inhibitor), and UTP (Ca^2+^ agonist and CaCC activator) of EDO recellularized ECM (f, *N* = 2) and BDO recellularized ECM (g, *N* = 2). After addition of Forskolin a small response in EDO samples was recorded, but not in the BDO samples. GlyH‐101 successfully blocked CFTR‐channel activity in both samples, as a decrease *I*_sc_ was recorded. Addition of 50 µM UTP showed an increase in current for both samples indicating the presence of CaCC. BDO, bile‐derived organoids; CaCC, calcium activated chloride channels; ECM, extracellular matrix; EDO, extrahepatic bile duct‐derived organoids; TEER, trans epithelial electrical resistance [Color figure can be viewed at wileyonlinelibrary.com]

Whole mount confocal analysis of ductal ECM recellularized with EDO or BDO after staining with acetylated α‐tubulin revealed presence of primary cilia in these samples (Figure 6a). The XZ‐plane revealed that cilia can be found on the apical side of the cells similar to the in vivo situation (Figure 6a, XZ plane). No acetylated α‐tubulin was detected in IDO‐repopulated scaffolds.

IDO recellularized samples were not assessed for functionality testing, as these organoids failed to fully repopulate the ductal ECM. The Ussing chamber experiments required larger ECM samples (L: 2 cm, W: 1 cm) and therefore the amount of cells used was increased fivefold (approx. 3.0 × 10^5^ cells per segment). Recellularization patterns were similar to those of the circular ductal ECM. However, due to the increased number of cells, EM had to be refreshed more often. During the last 7 days of the 21‐day period, medium was refreshed every 24 h.

Recellularized constructs had TEER of 17.8 Ω·cm^2^ (*SD*:±1.4 Ω·cm^2^) and 21.1 Ω·cm^2^ (*SD*:±4.7 Ω·cm^2^) for ductal ECM recellularized with EDO and BDO, respectively (Figure 6e). No fresh tissue was used as a control, due to the severity of denudation of EBD tissue. CFTR‐channel activity was induced by addition of forskolin (cAMP agonist). The segments repopulated with EDO showed a relatively small response (Figure 6f), whereas no change in *I*_sc_ was detected for BDO samples (Figure 6g). Both the EDO and BDO recellularized constructs responded to GlyH‐101 induced blockage of the CFTR‐channel with a reduction in anion secretion. This indicates that CFTR‐channels in the BDO recellularized ECM constructs were already active, presumably caused by the presence of forskolin in EM. Subsequent activation of CaCC with the purinergic Ca^2+^ agonist UTP caused a transient increase in *I*_sc_ in both samples. UTP was added to the luminal side of the constructs, thus the response indicates proper polarization of the epithelial cell layer with both CFTR and CaCC channels localized at the apical cell surface. Decellularized ECM was taken along as a negative control and did not respond to any of the additions (Figure S2).

## 4 DISCUSSION

The ability to engineer personalized functional EBD constructs in vitro may have considerable impact on the management of biliary complications after LT. Here, we demonstrated efficient decellularization of human EBD tissue and showed preservation of the ultrastructure of the EBD. Subsequent successful recellularization with cells derived from EDO and BDO, but not from IDO, was shown. The recellularized ductal ECM expressed mature cholangiocyte markers (such as KRT‐7, KRT‐19, NOTCH‐2, TFF‐1, and TFF‐2) and demonstrated cholangiocyte‐specific ion‐channel functionality. This provides proof of principle that in the near future, patient‐specific, transplantable and functional EBD tissue constructs could be engineered in vitro, which can be used to replace or repair damaged EBD tissue in vivo.

The use of decellularization strategies for ductal tissue engineering purposes has previously shown successful in animal models. Cheng et al. (2016) successfully transplanted a decellularized ureter, splinted by a silicone stent or a T‐tube, in guinea pigs. In parallel, Struecker et al. (2016) transplanted porcine abdominal aorta, which was recellularized with autologous cholangiocytes, in a porcine model. However, ureter or aorta ECM does not resemble the architecture of human EBD as these structures are lacking the complex PBG architecture.

Decellularization of human EBD tissue was efficient and did not damage the architecture of the ECM. The loss of connective tissue surrounding the EBD could account for the decrease in sGAG content, although this was not further quantified. The decrease of sGAG did not appear to impact the formation of confluent cell‐layers, as EDO and BDO successfully repopulated the surface of the ductal ECM. Further analysis of the repopulated ductal ECM showed cholangiocyte‐like cells, which expressed cholangiocyte markers on RNA and protein level. Furthermore, they had a functional barrier allowing measurements of vectoral transport of anions through cholangiocyte‐specific ion‐channels (CFTR and CaCC). These responses could not be compared with healthy and viable EBD‐tissue, as all EBD tissue obtained from LT procedures showed extensive denudation of the cholangiocyte monolayer and did not possess a functional barrier anymore. However, the responses measured were in similar order of magnitude as the responses measured for human gall bladder epithelium (Chinet et al., 1999).

Although IDO expressed similar cholangiocyte markers, they were less successful in fully repopulating the bile duct ECM. This difference in recellularization efficiency could be explained by regional differences in human biliary tissues. The extrahepatic and intrahepatic bile ducts are of different embryonic origin, arising from different progenitors during embryonic development (Zong & Stanger, 2011). This results in transcriptional differences between EBD or IBD cholangiocytes. Rimland et al. (2020) recently demonstrated that these differences are retained in vitro in the organoids initiated from different sources. Therefore, organoids of extrahepatic origin could be best suited to repopulate the decellularized ECM of the EBD. IDO, on the other hand, could potentially be more useful for repopulation of decellularized IBD (Willemse et al., 2020), which is vital for creating for functional liver tissue constructs in vitro.

Biliary organoids are an alternative source of primary cholangiocytes of which expansion in vitro is challenging (Tabibian et al., 2014). The organoids were obtained from healthy donors or from patient material (see Section 2 “*Sample procurement for organoid initiation*” for indications). No significant differences between healthy or “diseased” organoids were witnessed in manner of proliferation or mRNA expression profiles, and there was no difference in recellularization capacity. This indicates that autologous cells from patients could be used for personalized regenerative medicine purposes. BDO can obtained through less invasive ERCP procedures, making BDO more ideal for personalized purposes. EDO from donors could be used as an alternative when patient‐derived cells are not available.

Biliary LGR5^+^ organoids offer specific advantages when used in tissue engineering over other cell sources, such as induced pluripotent stem cells (iPSC), mostly because organoids are less prone to (epi)genetic variation (Huch et al., 2015; Rebuzzini et al., 2016). Furthermore, iPSC require extensive reprogramming and differentiation protocols, whereas no differentiation protocols were needed to create functional EBD constructs in vitro. The organoids already express mature cholangiocyte markers TFF‐1, TFF‐2, and MUC‐1 (MacParland et al., 2018; Segal et al., 2019). In addition, extrahepatic organoids have shown to be efficiently used for ductal tissue engineering purposes in vivo. Sampaziotis et al. (2017) demonstrated that extrahepatic organoids can successfully replace extrahepatic bile duct in mice, albeit that the culture medium for initiating and expanding the organoids differed from ours. Furthermore, they used an artificial collagen scaffold, which lacks tissue‐specific architecture, such as PGB. Therefore, the use of collagen scaffolds might be less optimal for long‐term homeostasis of the engineered bile duct. An advantage of applying decellularization strategies is that these architectural features do remain present after decellularization of EBD.

However, several “hurdles” have to be taken before decellularized and repopulated human ductal ECM can be clinically used. First and foremost, a translational step from the small two‐dimensional sections towards three‐dimensional (3D) tubular structures has to be made. This involves increasing the surface area that needs to be recellularized and an increase in the number of cells. Subsequently, there will be an increase in oxygen and nutrient consumption and it is likely that this translation requires more complex culture systems, such as perfusion‐based bioreactors. Furthermore, maintaining viability of cholangiocytes after transplantation would require the development of a blood vessel network. Struecker et al. (2016) showed feasibility of transplanting a bile duct construct solely with cholangiocytes in a large animal model without forming blood vessels (before transplantation). This could suggest that a preformed blood vessel network is not required, as formation of blood vessel after implantation appeared to be adequate. However, this study was performed in healthy animals and more research is required whether this holds true for patients with defective EBD tissue.

Another important issue is the use of non GMP‐compliant basement membrane extracts, such as Matrigel or Cultrex BME, for the initiation and expansion of organoids. These extracts are created from mouse tumor tissue (Benton et al., 2014; Hughes et al., 2010) and are limiting the clinical applications of the organoids and subsequently of recellularized EBD constructs (Willemse et al., 2017). To overcome this “hurdle,” clinically relevant and well‐defined culture substrates are required. Alternative candidates have already been investigated for organoids cultures (Giobbe et al., 2019; Gjorevski et al., 2016; Krüger et al., 2020).

## 5 CONCLUSION

Here, we show successful recellularization of decellularized EBD tissue using ductal organoids. Both EDO and BDO are promising cell sources to be used in personalized biliary tissue engineering, as they maintain cholangiocyte‐marker expression, showed restored barrier function and possessed cholangiocyte‐specific ion‐channel activity. In this study, we identified BDO as the most suitable candidate for future use in building functional 3D tubular EBD constructs. This is mostly due to easy access and minimally invasive collection of bile from patients.

## CONFLICT OF INTERESTS

The authors declare that there are no conflict of interests.

## AUTHOR CONTRIBUTIONS

Jorke Willemse, Jeroen de Jonge, and Monique M. A. Verstegen designed the study. Luc J. W. van der Laan, Monique M. A. Verstegen, and Jeroen de Jonge obtained funding. Jeroen de Jonge and Ivo J. Schurink obtained and surgically removed full length EBD from research livers. Jorke Willemse, Iris J. Voogt, and Ivo J. Schurink developed the decellularization protocol, performed the decellularization of EBD tissue, and characterized the ductal ECM. Floris J. M. Roos and Monique M. A. Verstegen initiated and expanded organoids. Iris J. Voogt created the recellularization procedure for EDO. Jorke Willemse and Floris J. M. Roos performed recellularization experiments with all three types of organoids. Jorke Willemse performed whole mount confocal analysis and RT‐qPCR experiments. Floris J. M. Roos, Marcel Bijvelds, and Hugo R. de Jonge performed the Ussing chamber experiments. Jorke Willemse, Floris J. M. Roos, Jeroen de Jonge, Iris J. Voogt, Ivo J. Schurink, Marcel Bijvelds, Hugo R. de Jonge, Luc J. W. van der Laan, and Monique M. A. Verstegen analyzed experimental data. Jorke Willemse and loris J. M. Roos drafted the figures. Jorke Willemse, Jeroen de Jonge, Monique M. A. Verstegen, and Floris J. M. Roos wrote the manuscript.

## Supporting information

Supplementary information.

Supplementary information.

Supplementary information.
