# Supplementary figures and images for: Scaffolds obtained from decellularized human extrahepatic bile ducts support organoids to establish functional biliary tissue in a dish
Source: Biotechnol Bioeng. 2020 Nov 9;118(2):836–51. doi: 10.1002/bit.27613 (PMC7894321; doi:10.1002/bit.27613)

# Supplementary figure S1


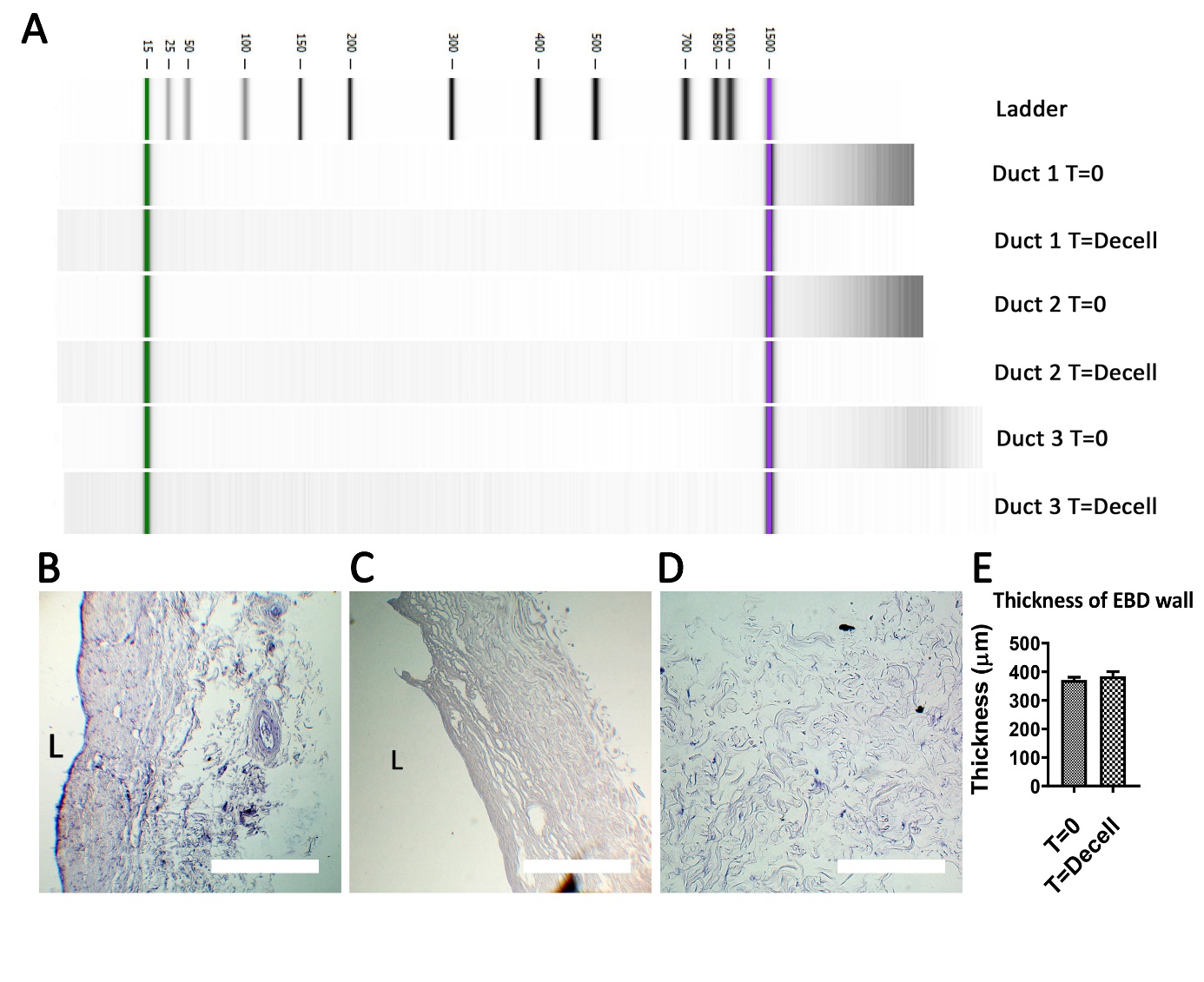


# Supplementary figure S2


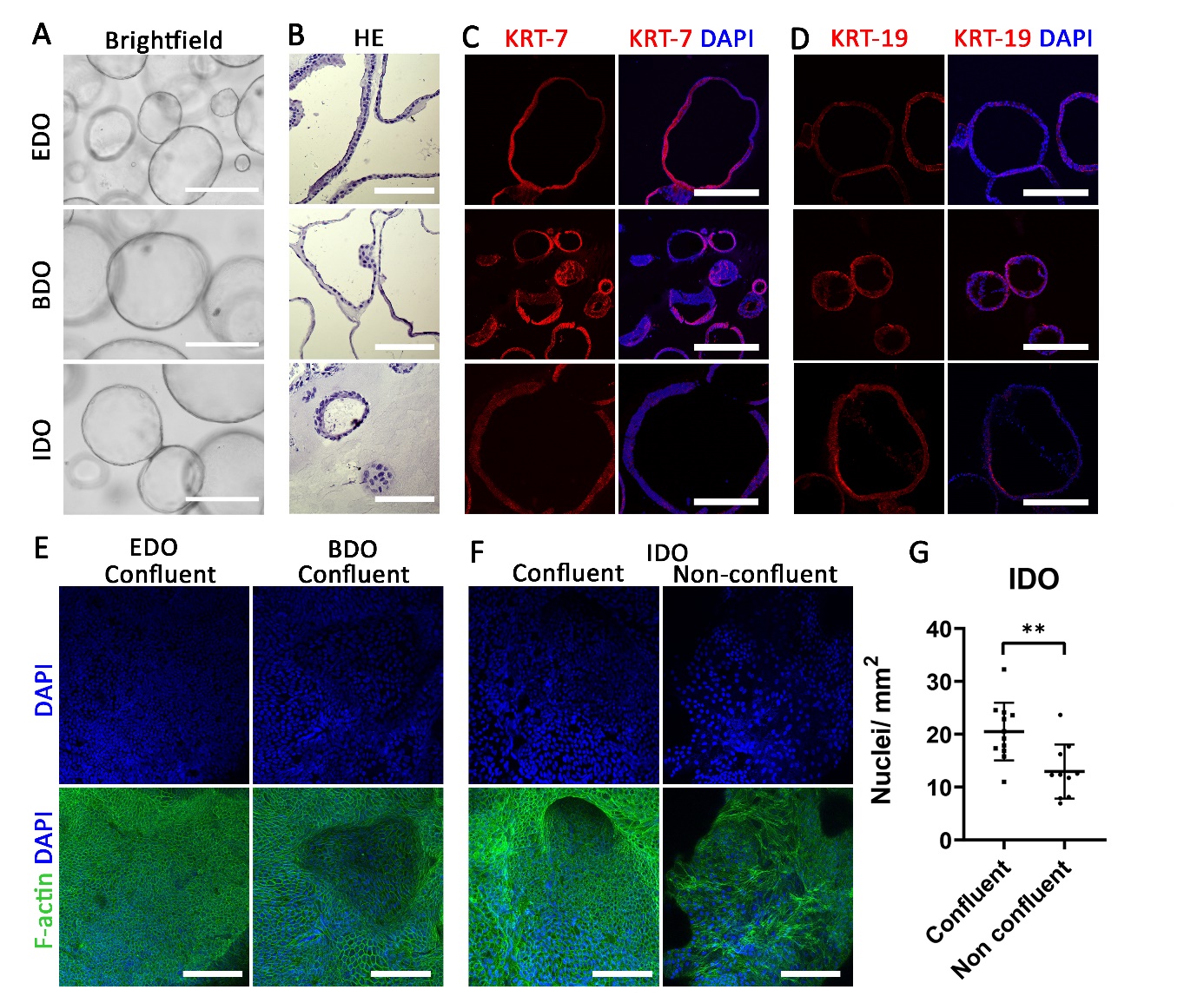


# Supplementary figure S3


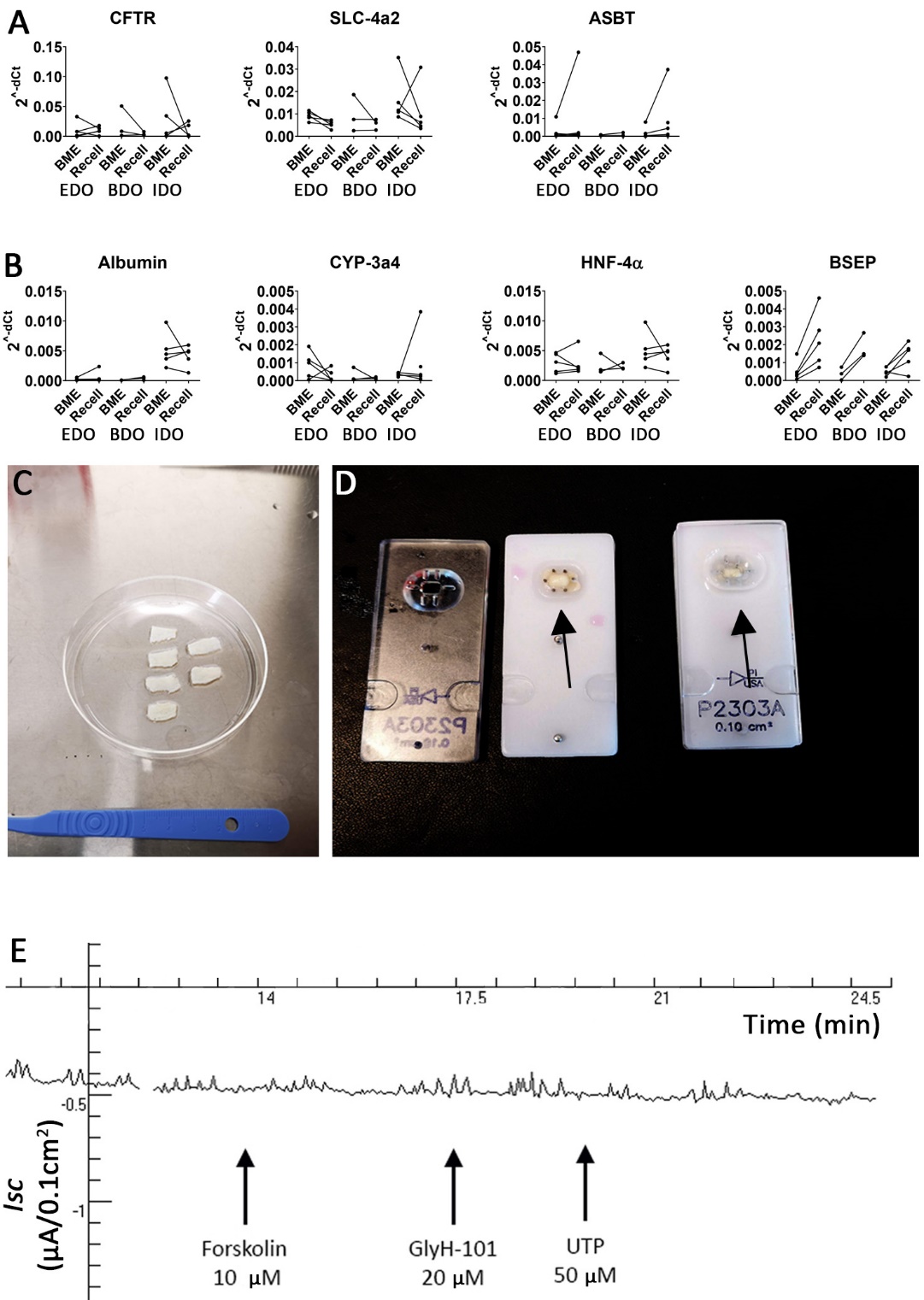

Supplement: Supplementary file 1 — Supplementary information. [file BIT-118-836-s001.docx]
