# Supplementary material for: Scaffolds obtained from decellularized human extrahepatic bile ducts support organoids to establish functional biliary tissue in a dish
Source: Biotechnol Bioeng. 2020 Nov 9;118(2):836–51. doi: 10.1002/bit.27613 (PMC7894321; doi:10.1002/bit.27613)
Supplement: Supplementary file 2 — Supplementary information. [file BIT-118-836-s002.docx]

# Supplementary info

Table 1: DNase solution

| Component | Concentration | Brand |
| --- | --- | --- |
| DNase 1 | 5U/L | Sigma |
| NaCl | 0.9% 100ml | Sigma |
| CaCl_2_ | 100mM | Sigma |
| MgCl_2_ | 100mM | Sigma |

Table 2: Medium supplement for Advanced DMEM/ F12

| Component | Amount | Concentration | Brand |
| --- | --- | --- | --- |
| Advanced DMEM/F12 | 500ml |  | Gibco |
| HEPES | 5ml | 1M | Life technologies |
| L-Glutamin | 5ml | 100X | Life technologies |
| Primocin | 1ml | 500mg/ml | Invivogen |
| Pen/Strep | 5ml | 10000 U/ml | Life technologies |

Table 3: Medium formulation for Start Up Medium (SEM) and Expansion Medium (EM). Medium components with a * are only added to SEM.

| Component | Concentration | Brand |
| --- | --- | --- |
| Adv+ |  | Gibco |
| N2 | 1% | Gibco |
| B27 | 2% | Gibco |
| N-Acetylcystein | 1,25 mM | Sigma |
| gastrin | 10 nM | Sigma |
| EGF | 50 ng/ml | Peprotech |
| FGF10 | 100 ng/ml | Peprotech |
| HGF | 25 ng/ml | Peprotech |
| nicotinamide | 10nM | Sigma |
| A83.01 | 5 µM | Tocris |
| Forskolin | 10 µM | Torcris |
| R-Spondin | 10% | Conditioned medium |
| WNT* | 30% Wnt | Conditioned medium |
| Noggin* | 25 ng/ml | Conditioned medium |
| Y27632* | 10µM | Tocris |
| hES cell cloning recovery solution* | 1:1000 dilution | Stemgent |

Table 4: List of primary antibodies used for Immunohistochemistry (IHC) or Whole mount confocal. Antibodies with ** are only used for whole mount confocal.

| Primary antibody | Raised in | Dilution | Supplier |
| --- | --- | --- | --- |
| Collagen Type I | Rabbit | 1:60 | Novus biologicals |
| Collagen Type IV | Rabbit | 1:50 | Novus biologicals |
| Acetylated α-tubulin** | Mouse | 1:100 | Sigma |
| ZO1** | Rabbit | 1:100 | Proteintech |
| KRT 7** | Mouse | 1:100 | Dako |
| KRT 19** | Mouse | 1:100 | Dako |

Table 5: List of fluorescent labeled secondary antibodies

| Secondary antibody | Raised in | Against | Dilution | Supplier |
| --- | --- | --- | --- | --- |
| Alexa 555 | Goat | Mouse | 1:100 | Fisher scientific |
| Alexa 488 | Goat | Rabbit | 1:100 | Fisher scientific |

Table 6: List of qPCR primers

| Primer | Forward sequence 5'to 3' | Reverse sequence 5'to 3' |
| --- | --- | --- |
| GAPDH | CTTTTGCGTCGCCAGCCGAG | CCAGGCGCCCAATACGACCA |
| HPRT-1 | ACCAGTCAACAGGGGACATAA | CTTCGTGGGGTCCTTTTCACC |
| B2M | GTGTCTGGGTTTCATCCATC | GGCAGGCATACTCATCTTTT |
| LGR-5 | GTCAGCTGCTCCCGAATCCC | TGAAACAGCTTGGGGGCACA |
| KRT-7 | GGGGACGACCTCCGGAATAC | CTTGGCACGCTGGTTCTTGA |
| KRT-19 | GCACTACAGCCACTACTACACGA | CTCATGCGCAGAGCCTGTT |
| EPCAM | GACTTTTGCCGCAGCTCAGGA | AGCAGTTTACGGCCAGCTTGT |
| TROP-2 | CGAGCTTGTAGGTACCCGGCG | TGCGCCGAGGAATCAGGAAGC |
| SOX-9 | ACCAGTACCCGCACTTGCAC | GCGCCTTGAAGATGGCGTTG |
| HNF-1β | TCACAGATACCAGCAGCATCAGT | GGGCATCACCAGGCTTGTA |
| NOTCH-2 | CATCTGGATGGGCTGGTGCC | AGGATGATTTCATACCCCGAGTGC |
| TTF-1 | ACAAGCTGCTGTACACGGACA | AAGTTTCCAGGGCCGGGCAAT |
| TTF-2 | TCTGTCCTGCCTCCCTGATCCA | CTCTGGCACGTGAATCCCGGT |
| MUC-1 | CTGTCAGTGCCGCCGAAAGA | CGTGCCCCTACAAGTTGGCA |
| KI-67 | CTACGGATTATACCTGGCCTTCC | AGGAAGCTGGATACGGATGTCA |
| Vimentin | CGGGAGAAATTGCAGGAGG | TGCTGTTCCTGAATCTGAGC |
| SLC-4a2 | GAAGATTCCTGAGAATGCCG | GTCCATGTTGGCACTACTCG |
| AQP-1 | GGCCAGCGAGTTCAAGAAGAA | TCACACCATCAGCCAGGTCAT |
| CFTR | TGGCGGTCACTCGGCAATTT | TCCAGCAACCGCCAACAACT |
| ASBT | GGTGGCCTTTGACATCCTCCC | GCATCATTCCGAGGGCAAGC |
| BSEP | TGAGCCTGGTCATCTTGTG | TCCGTAAATATTGGCTTTCTG |
| Albumin | CTGCCTGCCTGTTGCCAAAGC | GGCAAGGTCCGCCCTGTCATC |
| CYP-3a4 | AGCAAAGAGCAACACAGAGCTGAA | CAGAGGTGTGGGCCCTGGAAT |
| HNF-4α | GTACTCCTGCAGATTTAGCC | CTGTCCTCATAGCTTGACCT |

Table 7: Composition of Meyler’s medium used in the Ussing chambers

| Concentration | Compound |
| --- | --- |
| 128mM | NaCl |
| 4.7mM | KCl |
| 1.3mM | CaCl_2_ |
| 1mM | MgCl_2_ |
| 0.3mM | Na_2_HPO_4_ |
| 0.4mM | NaH_2_PO_4_ |
| 20mM | NaHCO_3_ |
| 10mM | HEPES |
