# Supplementary material for: Scaffolds obtained from decellularized human extrahepatic bile ducts support organoids to establish functional biliary tissue in a dish
Source: Biotechnol Bioeng. 2020 Nov 9;118(2):836–51. doi: 10.1002/bit.27613 (PMC7894321; doi:10.1002/bit.27613)
Supplement: Supplementary file 3 — Supplementary information. [file BIT-118-836-s003.docx]

# Supplementary figures

**Supplementary figure S1: A: No double stranded DNA was found after decellularization as shown by the results of the BioAnalyzer. For this, paired samples (n=3) before (T=0) and after decellularization (T=decell) were analyzed. Histological analysis by HE staining of a bile duct sample before decellularization (T=0) (B) and after decellularization (T=Decell) (C). L indicate the luminal side of the EBD. Scale bars: 400µm. D: HE staining of loose connective tissue that was removed from the EBD during the decellularization procedure. The connective tissue did not contain blood vessels, muscle fibers and/or PBG. Scale bar: 100µm. E: The decellularization procedure did not affect the thickness of the EBD wall (P=0.856) as was measured on HE-sections of before decellularized EBD samples (N=6 different samples per condition, N=10 independent measurements per sample).**

Supplementary figure S2: A: Bright field pictures of EDO, BDO and IDO grown in BME. Organoids from all three sources are spherical in shape. Scale bars: 400µm. B: HE images of EDO (top), BDO (middle) and IDO (bottom) grown in BME. Scale bars: 400µm. KRT-7 (C) and KRT-19 (D) staining of EDO (top), BDO (middle) and IDO (bottom) of organoids cultured in BME show that organoids are KRT-7 and KRT-19 positive. Scale bars: 200µm. EF: Whole mount confocal images after recellularization of the decellularized ECM with EDO (E, left column), BDO, (E, right column) and IDO (F). EDO and BDO samples were completely confluent and had the highest nuclear density. F-actin + DAPI staining revealed ‘honey comb’-like structures on the surface of the repopulated ECM. Image representative for N=4 independent bile duct scaffolds recellularized with N=5 EDO and N=3 BDO lines. Scale bars: 100µm. F: IDO were less capable of fully repopulating the surface of the decellularized ECM, as DAPI staining revealed differences in nuclear density between confluent (left column) and non-confluent (right column). ‘Honey comb’-like structures are seen in confluent samples, but not in non-confluent samples. Scale bars: 100µm. G: There is a significant difference (**, P<0.01) between the number of nuclei per mm^2^ grown for IDO repopulated scaffolds. Fully repopulated (confluent) samples (N=2 for IDO) yielded 20.5 nuclei per mm^2^ (SD:+-5.2), whereas the nuclear density was 12.9 nuclei per mm^2^ (SD:+-4.8) for the non-confluent samples (N=3 for IDO).

**Supplementary figure S3: AB: Gene expression analysis (RT-qPCR) of recellularized ECM for cholangiocyte-related genes (CFTR, SLC-4a2 and ASBT) (A) and hepatocyte-related genes (Albumin, CYP-3a4, HNF-4α and BSEP) (B). The RT-qPCR data is displayed as 2-dCt and each recellularized sample (after) is connected to a matched BME control (before). No significant differences were found in the expression of these cholangiocyte (CFTR, SLA-4a2 and ASBT) and hepatocyte (Alb, Cyp3A4, HNF4a and BSEP) genes, according to the Wilcoxon signed rank test. C: Larger ductal ECM scaffolds (L:2cm, W: 1cm) (C) were used to assess the TEER and vectoral Ion-transport by CFTR functionality in the repopulated bile ducts using an Ussing chamber set up. D: the recellularized scaffolds were placed inside the Ussing chamber holders. The black arrows indicate the scaffolds, which were placed over a set of needles to hold them in place. E: The Ussing chamber results of decellularized ECM negative control (without cells) (N=1). No response to added components was detected.**

Supplementary information (tables)

Table 1: DNase solution

Table 2: Medium supplement for Advanced DMEM/ F12

Table 3: Medium formulation for Start Up Medium (SEM) and Expansion Medium (EM). Medium components with a * are only added to SEM.

Table 4: List of primary antibodies used for Immunohistochemistry (IHC) or Whole mount confocal. Antibodies with ** are only used for whole mount confocal.

Table 5: List of fluorescent labeled secondary antibodies

Table 6: List of qPCR primers

Table 7: Composition of Meyler’s medium used in the Ussing chambers
